# CD14^high^CD16^+^ monocytes are the main producers of Interleukin-10 following clinical heart transplantation

**DOI:** 10.3389/fimmu.2023.1257526

**Published:** 2023-10-23

**Authors:** Kristina Ludwig, Evgeny Chichelnitskiy, Jenny F. Kühne, Bettina Wiegmann, Jasper Iske, Nadine Ledwoch, Fabio Ius, Kerstin Beushausen, Jana Keil, Susanne Iordanidis, Sebastian V. Rojas, Jawad Salman, Ann-Kathrin Knoefel, Axel Haverich, Gregor Warnecke, Christine S. Falk

**Affiliations:** ^1^ Institute of Transplant Immunology, Hannover Medical School, Hannover, Germany; ^2^ Department of Surgery, Charité-Universitätsmedizin Berlin, Corporate Member of Freie Universität Berlin and Humboldt-Universität zu Berlin, Berlin, Germany; ^3^ Department for Cardiothoracic, Transplantation and Vascular Surgery, Hannover Medical School, Hannover, Germany; ^4^ Lower Saxony Center for Biomedical Engineering, Implant Research and Development, Hannover Medical School, Hannover, Germany; ^5^ DZL, German Center for Lung Diseases, BREATH site, Hannover, Germany; ^6^ Department of Cardiothoracic and Vascular Surgery, Deutsches Herzzentrum der Charité, Berlin, Germany; ^7^ Heart and Diabetes Center Nordrhein-Westfalen, University Hospital Ruhr-University Bochum, Bad Oeynhausen, Germany; ^8^ Department of Cardiac Surgery, University Hospital Heidelberg, Heidelberg, Germany; ^9^ DZIF, German Center for Infection Research, TTU-IICH, Hannover, Germany

**Keywords:** heart transplantation, organ transplantation, ischemia-reperfusion injury, interleukin-10, monocytes

## Abstract

**Introduction:**

Following heart transplantation, a cascade of immunological responses is initiated influencing the clinical outcome and long-term survival of the transplanted patients. The anti-inflammatory cytokine interleukin-10 (IL-10) was shown to be elevated in the blood of heart transplant recipients directly after transplantation but the releasing cell populations and the composition of lymphocyte subsets following transplantation have not been thoroughly studied.

**Methods:**

We identified immune cells by immunophenotyping and analyzed intracellular IL-10 production in peripheral blood mononuclear cells (PBMC) of heart transplanted patients (n= 17) before, directly after and 24h post heart transplantation. The cells were stimulated with lipopolysaccharide or PMA/Ionomycin to enhance cytokine production within leukocytes *in vitro*.

**Results and discussion:**

We demonstrate that intermediate monocytes (CD14^high^CD16^+^), but not CD8^+^ T cells, CD4^+^ T cells, CD56^+^ NK cells or CD20^+^ B cells appeared to be the major IL-10 producers within patients PBMC following heart transplantation. Consequently, the absolute monocyte count and the ratio of intermediate monocytes to classical monocytes (CD14^+^CD16^-^) were specifically increased in comparison to pre transplant levels. Hence, this population of monocytes, which has not been in the focus of heart transplantation so far, may be an important modulator of clinical outcome and long-term survival of heart transplant recipients. Alteration of blood-circulating monocytes towards a CD14^high^CD16^+^ phenotype could therefore shift the pro-inflammatory immune response towards induction of graft tolerance, and may pave the way for the optimization of immunosuppression.

## 1 Introduction

Multiple cardiac diseases, like congenital and acquired cardiomyopathies ultimately lead to end stage heart failure for which to date there is no curative treatment other than heart transplantation (HTx). Besides the challenges accompanied by the transplantation process itself, i.e. shortage of donor hearts, logistic requirements, and the curative potential the surgery inherits, it is a vital endeavor to aim for the best possible clinical outcome. Hence, the allograft should be prevented from acute and/or chronic rejection thereby contributing to both graft and patient survival.

Cold storage of the donor heart before transplantation followed by its reconnection to the recipient’s circulatory system intraoperatively leads to ischemia-reperfusion injury (IRI). This inflammatory process is associated with hypoxia, activation of the endothelium and immune cells as well as the production of pro-inflammatory cytokines/chemokines that eventually contribute to primary graft dysfunction and acute or chronic rejection (1–4). The immediate immune response is induced through ligand binding to unspecific receptors on the surface of innate immune cells e.g. bacterial membrane component lipopolysaccharide (LPS) to Toll-like receptor (TLR) 4 on monocytes and neutrophils or through missing-self activation on NK cells. Subsequent priming and trafficking of T cells is induced by crosslinking of the T cell receptor via an MHC/antigen complex. Triggering specific signaling pathways *in vitro* could model selective cellular immune responses and reveal particular differences in the functional state of these cells at different time points after HTx.

Coherent with the immune responses following IRI is the secretion of multiple cytokines, a group of proteins secreted by various activated cell types exerting diverse effector functions. Cytokines play an important role not only for modulation of both chronic and acute inflammation, but also for suppression of pro-inflammatory states and induction of tissue recovery. Interleukin-10 (IL-10) is a cytokine produced by almost all leukocytes in response to different stimuli. Binding to the ubiquitously expressed IL-10 receptor alpha/beta heterodimer mediates anti-inflammatory responses e.g. suppression of pro-inflammatory cytokines, downregulation of co-stimulatory molecules such as CD86 and blocking of CD28-B7 interactions resulting in dampening of T cell activation and thereby prevention of inadequate immune responses (5, 6). Previous own findings detected a significant increase of the anti-inflammatory cytokine IL-10 in patient plasma directly after HTx (7). However, it is hitherto unknown, which circulating cell population may be responsible for the peak in IL-10 detected in HTx patient plasma.

A better understanding of the cellular source of IL-10 and the associated cytokine network would not only allow for the precise and selective usage of immunosuppression, but also allow shifting the equilibration of cytokines/chemokines after HTx to an anti-inflammatory state. While a network of numerous cytokines and chemokines precisely determine immunological processes, their secretion strongly depends on the dynamics of immune cell types after implantation of the donor organ. Thus, in this study we provide insights into leukocyte dynamics and cellular processes occurring in recipient blood following HTx. We further identified CD14^high^CD16^+^ monocytes as the cellular source of IL-10 in HTx patient plasma. Those findings might enable improved clinical control of the consequences of IRI and the pro-inflammatory milieu it triggers.

## 2 Materials and methods

### 2.1 Patients and sample collection

The study cohort consisted of 17 patients with underlying terminal heart failure that underwent HTx at the Department of Cardiothoracic Transplantation and Vascular Surgery at Hannover Medical School, Hannover, Germany (Ethical approval 7913_BO_S_2018). Demographic data of HTx recipients are shown in **Table 1**; only one patient developed histopathological proven rejection within the first month following HTx. Donor hearts were preserved by two techniques: Standard of care (SOC) donor hearts were perfused with cardioplegic solution, Histidine-Tryptophan-Ketoglutarate (HTK), followed by cold storage at 4°C after explanation until transplantation into the recipient. *Ex-situ* heart perfusion (ESHP) donor hearts were also temporary perfused with HTK solution before being continuously oxygenated and perfused with both perfusion solution and donor blood within an organ care system (OCS) device (TransMedics, Andover, USA) at 37°C until transplantation. Standard immunosuppressive therapy was started intraoperatively including steroids, mycophenolate mofetil (MMF) and calcineurin inhibitors. Severe primary graft dysfunction (PGD) was classified according to ISHLT Consensus Statement, i.e. patients with PGD requiring veno-arterial ECMO (8).

**Table 1:** Baseline demographics.

Characteristics	Study cohort (n=17)	TruCount (n=13)	ICS (n=11)	RT-PCR (n=9)
Donor				
**Age at donation, median, years**	40/ (10-59)	41/ (27-59)	47/ (10-59)	48/ (10-59)
**Sex, n/%**	F 7/ (41%) M 10/ (59%)	F 5/ (38%) M 8/ (62%)	F 4/ (36%) M 7/ (64%)	F 4/ (44%) M 5/ (56%)
Recipient				
**Age at HTx, median, years**	52 (9-68)	52 (26-66)	57 (9-68)	57 (9-68)
**Sex, n/%**	F 4/ (24%)	F 2/ (15%)	F 2/ (18%)	F 2/ (22%)
	M 13/ (76%)	F 2/ (1M 11/ (85%)	M 9/ (82%)	M 7/ (78%)
**Severe PGD**	4/ (24%)	3/ (23%)	3/ (27%)	3/ (33%)
Transplant indication				
** DCM**	4/ (24%)	4/ (31%)	2/ (18%)	1/ (11%)
** ICM**	2/ (12%)	1/ (8%)	1/ (9%)	1/ (11%)
** others**	11/ (64%)	8/ (61%)	8/ (73%)	7/ (78%)
**CCT, min ± SD**	370 ± 80	388 ± 58	344 ± 81	338 ± 90
**CIT, min ± SD**	142 ± 56	116 ± 30	150 ± 66	158 ± 71
**Previous LVAD implantation n/%**	14/ (82%)	11/ (69%)	9/ (82%)	7/ (78%)
Treatment group, n/%				
** SOC**	5/ (29%)	2/ (15%)	4/ (36%)	3/ (33%)
** ESHP**	12/ (71%)	11/ (85%)	7/ (64%)	6/ (67%)
Biopsy-proven rejection				
** 1R, n/%**	2/ (12%)	1/ (8%)	1/ (9%)	1/ (11%)

CCT, cross-clamp time; CIT, cold ischemic time; DCM, dilated cardiomyopathy; ESHP, Ex-situ heart perfusion; F, female; HTx, heart transplantation; ICM, ischemic cardiomyopathy; ICS, intracellular staining; LVAD, left ventricular assist device; M, male; PGD, primary graft dysfunction; 1R, histopathological mild rejection; SOC, standard of care.

### 2.2 Blood sample collection and processing

Blood samples were collected from HTx recipients preoperatively (*pre*), 2-4 hours (*T0*), 24 hours (*T24*) and 3 weeks after transplantation (*3wk*) (**Supplementary Figure 1**). Whole blood was used for quantification of lymphocytes using BD TruCount^®^. For quantification of cytokine/chemokines, plasma was isolated by centrifugation at room temperature at 2100 rpm for 15min. Supernatants were frozen at -20°C until further usage for multiplex cytokine analysis. The cellular components of the blood were resuspended in phosphate-buffered saline (PBS; Invitrogen, Carlsbad, USA) and peripheral blood mononuclear cells (PBMC) were isolated via Biocoll gradients (Biochrom, Berlin, Germany) according to manufacturer’s instructions. PBMC were frozen in RPMI (Invitrogen) supplemented with 50% fetal calf serum (FCS; Invitrogen) and 10% dimethyl sulfoxide (DMSO; Carl Roth, Karlsruhe, Germany), stored at -80C° and liquid nitrogen, respectively.

### 2.3 Cytokine and chemokine quantification

The concentrations of 50 cytokine/chemokine proteins in the plasma of HTx patients (n=17) were measured for time points *pre*, *T0* and *T24* using Luminex-based multiplex technique (27 Plex, 23 Plex, Bio-Rad, Hercules, USA) according to manufacturer’s instructions. Bio-Plex Manager 6.1 software (Bio-Rad) was used to calculate standard curves and concentrations.

### 2.4 Leukocyte cell count analyses

Absolute numbers of leukocytes, including CD3^+^ T cells with a focus on CD4^+^ T cells, CD8^+^ T cells, naïve CD4^-^CD8^-^ T cells, CD19^+^ B cells, CD56^+^ NK cells, monocytes and granulocytes in whole-blood samples (n=13) for the time points *pre*, *T0*, *T24* and *3wk* post HTx were determined using BD TruCount^®^ beads according to manufacturer’s instructions (BD Biosciences, Franklin Lakes, USA). Samples were acquired with a LSR-II flow cytometer (BD Biosciences); immune cell subsets were gated using Diva software (Version 8.0.1, BD Biosciences).

### 2.5 *In-vitro* PBMC stimulation for induction of IL-10

For stimulation of PBMC from HTx patients (n=11) at *pre*, *T0* and *T24* time points, cells were resuspended in RPMI–1640 medium with 10% FCS, 2 mM L-glutamine, 100 U/ml penicillin/streptomycin, 1 mM sodium pyruvate (all Invitrogen) at 2 x 10^6^ cells/ml. To analyze cell type specific IL-10 and IFN-γ expression, cells were stimulated with either LPS (0,5 µg/ml, Sigma-Aldrich, St. Louis, USA) or Phorbol-12-myristate-13-acetate (PMA, 50 ng/ml, Merck-Millipore, Darmstadt, Germany) and Ionomycin (2,5 µg/ml, Merck-Millipore) (PMA/Iono) for 15h at 37°C and 5% CO_2_. Unstimulated PBMC in media (RPMI-1640 plus supplements) served as negative control. Brefeldin A (BFA, BioLegend, San Diego, USA), was added after 2h stimulation and cells were incubated for another 13h at 37°C and 5% CO_2_, resulting in 15h total stimulation time. Of note, BFA was only added to the cells, which were later used for flow cytometry analyses.

### 2.6 Intracellular cytokine staining and flow cytometry

All steps were performed as recommended by the guidelines for the use of flow cytometry in immunological studies (9). After stimulation and washing of *in vitro* stimulated PBMC with PBS (Invitrogen) cells were stained with cell viability dye (LIVE/DEAD Yellow fluorescent reactive dye, ThermoFisher Scientific, Waltham, USA) according to manufacturer’s instructions for 15min at room temperature. Next, cell surface staining using fluorescently labelled marker specific antibodies (Abs; **Supplementary Table 1**) for 30min at 4C° was accomplished, washing with PBS, fixing and permeabilizing using the Fix/Perm kit (ThermoFisher Scientific) according to manufacturer’s instructions. For intracellular staining (ICS), IL-10 and IFN-γ monoclonal Abs were applied together with appropriate isotype control (**Table S1**) for another 30min at 4C°. Cells were acquired with an LSR-II flow cytometer (BD Biosciences) and data were analyzed using Diva software (Version 8.0.1, BD Biosciences).

### 2.7 RNA isolation and real-time PCR

Total RNA was isolated from PBMC of HTx patients (n = 9) using EXTRAzol RNA isolation kit (DNA Gdansk, Blirt S.A., Danzig, Poland) according to manufacturer’s instructions. RNA was dissolved in RNAse-free H_2_O and RNA concentration was determined using Nanodrop (ThermoFisher Scientific). 750 ng RNA was applied for cDNA synthesis using RevertAid H Minus First Strand cDNA Synthesis Kit (ThermoFisher Scientific). Real time polymerase chase reaction (RT-PCR) was performed with LightCycler96 (Roche, Basel, Switzerland) utilizing FastStart essential DNA probes Master (Roche) and Taqman Gene Expression Assays (ThermoFisher Scientific) for the following genes: Glyceraldehyde-3-phosphate dehydrogenase (GAPDH, Hs99999905_m1), IL-10 (Hs00173499_m1) and IFN-γ (Hs0098921_m1). Target mRNA expression is presented as fold induction (2^-ΔΔCt^) compared to unstimulated samples at time point *pre* and the reference gene GAPDH. Data were acquired and analyzed using LightCycler Software Version 1.1.0.1320 (Roche).

### 2.8 Statistical analyses

D’Agostino-Pearson omnibus normality test was used to test data for normality and data distribution with statistical analyses were performed as stated in figure legends. P values of <.05 were considered statistically significant with gradations as indicated in the figure legends. All statistical analyses were performed using GraphPad Software Prism (Version 7, La Jolla, CA). The association of IL-10 concentrations with clinical parameters was assessed using a linear model presented as a Forest-plot using the R-Studio (R Version: R-4.0.3) and the packages: “forestmodel” and “tidy verse”. For statistical analysis of IL-10 kinetic plot the packages “ggplot2”, “ggpubr”, “shapirotest”, “Rstatix”, and “dunn.test” were used.

### 2.9 Unsupervised cluster and principal component analyses

The cytokine/chemokine protein dataset was analyzed using Qlucore Omics Explorer (Version 3.7, Lund, Sweden). Data were log_2_ transformed, scaled to mean zero, variable one, and threshold of 0.01. Discriminating variables were determined using linear models and multigroup analysis of variance (ANOVA) comparison and hierarchical clustering and PCA were performed.

## 3 Results

### 3.1 Plasma cytokine patterns in HTx recipients are distinct before and after transplantation and IL-10 correlates with PGD

**Figure 1:**
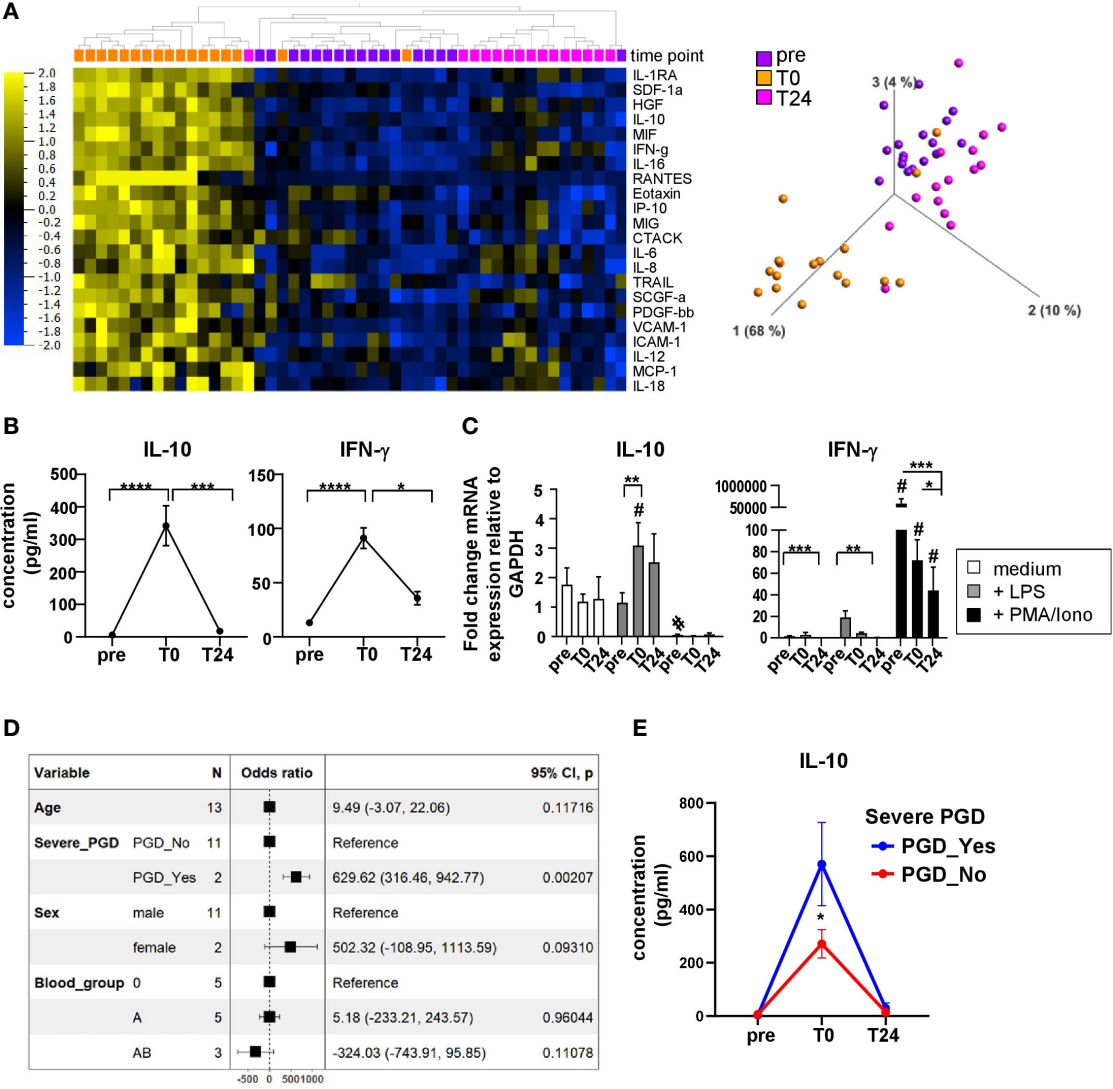
Distinct immunomodulatory plasma profile in heart recipients directly post HTx. **(A)** Heatmap displaying multigroup comparison of soluble immune mediators quantified in n=17 HTx recipient plasma using Luminex-based multiplex assay with the time points *pre*, *T0* and *T24* set as variables. With a q-value cut-off of 0.002 and p-value cut-off at 0.001, 22 cytokines were found significantly different among the groups. Subsets are ordered according to increasing q-values. Data are organized by unsupervised hierarchical clustering. Blue color indicates lower expression, yellow color indicates higher expression. Principal Component Analysis (PCA) including the 22 subsets visualizing the data set in a three-dimensional space. **(B)** Cytokine concentrations for IL-10 and IFN-γ in patient plasma before and after HTx. Data were tested for normality and 1-way-Anova was calculated for n=17 HTx patients; data are shown as mean ± SEM. **(C)** RT-PCR analysis for unstimulated, LPS or PMA/Ionomycin stimulated PBMC at time points *pre*, *T0* and *T24* from n=9 patients. Fold induction is set in relation to GAPDH expression. Data were tested for normality and 2-way-ANOVA for normal distributed data was performed for IL-10 RT-PCR data; RM-one-way-ANOVA was calculated for non normal-distributed IFN-γ RT-PCR data. B, C: Significances are shown with gradations: p ≤ 0.05 (*), p ≤ 0.005 (**), p ≤ 0.0005 (***), p ≤ 0.0001 (****); # is shown if stimulation differs from unstimulated at corresponding time point. **(D)** The linear model to assess the association of IL-10 concentrations with clinical parameters such as age, severe PGD, sex, and blood group at time point: T0. A Forest-plot is presented. The model fit R2 = 0.818 (adj. R2 = 0.562) **(E)** IL-10 kinetic over all time points: pre, T0, T24 split by the presence of severe PGD. The data’s normality was assessed using the Shapiro test, and the pairwise comparison was done using the Dunn test. Asterisk indicate: *p<0.05.

Both surgical intervention and IRI after solid organ transplantation result in humoral and cellular immune responses in the recipient, contributing to the cytokine/chemokine microenvironment critical for allograft acceptance or rejection. To execute their effector functions, immune cells are capable of producing cytokines and chemokines to navigate cells during inflammation and tissue-repairing processes, to mediate cell homeostasis and migration into tissues. In our current study, 50 soluble immune mediators were quantified in patient plasma at different time points following HTx (**Supplementary Figure 1**) and analyzed by applying multiple comparison statistics. We identified 22 soluble proteins with significantly discriminating values before (*pre)*, and after HTx at *T0* and *T24* (**Figure 1A**). Principal component analysis (PCA) revealed a combined clustering of the time points *pre* and *T24* while clearly separating plasma samples at *T0*. This implies a transient alteration of the cytokine and chemokine microenvironment at *T0*, most likely because of IRI, and furthermore confirms our previous findings (7). Representative for both anti- and pro-inflammatory cytokines, IL-10 and IFN-γ were part of the unsupervised clustering and PCA. Notably, their plasma concentrations showed a distinct peak at *T0* (**Figure 1B**). When analyzing the mRNA expression of IL-10 and IFN-γ by PBMC upon *in vitro* stimulation, basal expression of IL-10 mRNA was detected at all time points (*pre*, *T0* and *T24*) and could be enhanced by LPS stimulation at *T0* and *T24* (**Figure 1C**). Of note, IL-10 mRNA was not induced by stimulation with PMA/Ionomycin. The selective IL-10 mRNA expression by LPS may derive from monocytes, but not T or NK cells, contained in the bulk PBMC samples, as it primarily activates monocytes via LPS–TLR4 interaction.

Interestingly, IFN-γ mRNA was almost not detectable in unstimulated and LPS-stimulated PBMC (except for *pre*). By contrast, stimulation with PMA/Ionomycin induced highest IFN-γ mRNA expression levels at all time points that significantly decreased at *pre* vs *T0* and *T24* (**Figure 1C**). This effect may be due to the immunosuppression (IS) initiated intraoperatively which consequently impairs cytokine production by leukocytes.

Finally, we also tested a potential association between clinical parameters such as severe PGD, but also sex, age, and blood group and plasma cytokines, i.e. IL-10 concentrations at time point T0 (**Figure 1D**). We found that patients developing severe PGD, but no other clinical parameter tested, showed a strong positive association with IL-10 concentration, as assessed using a linear model and presented as a Forest-plot. The statistically significant differences in IL-10 concentrations between patients with and without severe PGD at time point T0 were also confirmed in kinetic analysis (**Figure 1E**). This association between PGD and IL-10, an anti-inflammatory cytokine, seems to be counterintuitive and, hence, may represent a counter-regulatory loop triggering pro and anti-inflammatory cytokines in patients with PGD. The effect of PGD on cytokine and chemokine secretion was defined by us previously (7).

### 3.2 HTx induced dynamic changes in lymphocyte and monocyte compositions

**Figure 2:**
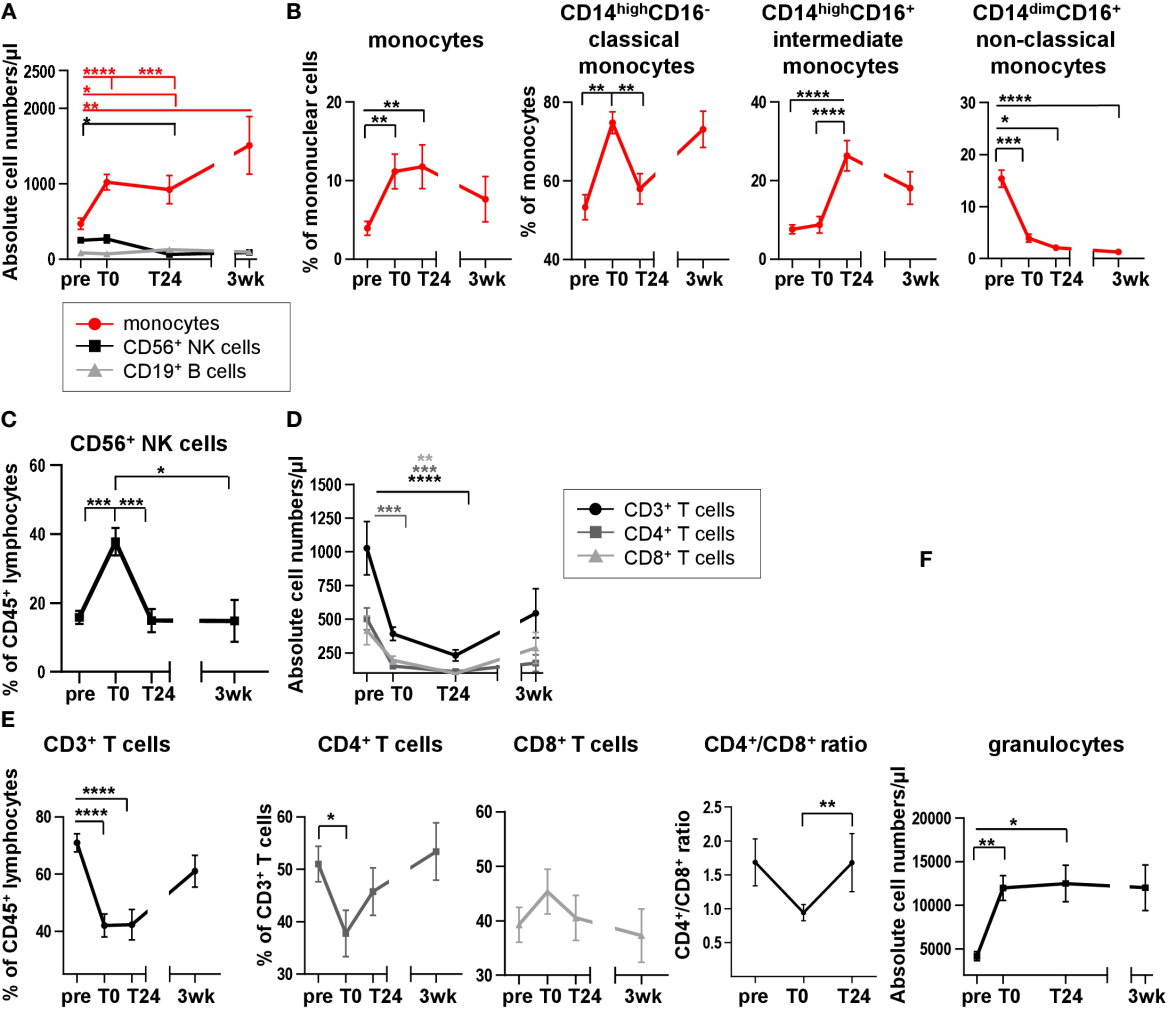
Analysis of leukocyte distribution and subpopulations reveals dynamic changes in peripheral blood lymphocyte and monocyte compositions directly after HTx. **(A)** Absolute cell numbers of monocytes, CD56^+^ NK cells and CD19^+^ B cells were quantified in whole blood samples from 13 HTx patients using TruCount® analyses. **(B)** Flow cytometric analysis of cell surface stained PBMC from 17 HTx recipients for monocytes with subsequent analysis of CD14^+^CD16^-^, CD14^+^CD16^+^ and CD14^low^CD16^+^ monocyte subsets at different time points pre and post HTx; gating as shown in **Supplementary Figure 2B**. **(C)** Frequencies of CD56^+^ NK cells at different time points pre and post HTx were determined by flow cytometry in PBMC from 17 patients. Gating as shown in **Supplementary Figure 1B**. **(D)** Absolute cell numbers of CD3^+^ T cells, CD4^+^ T cells and CD8^+^ T cells were quantified in whole blood samples from 13 HTx patients using TruCount® analyses. **(E)** Frequencies of CD3^+^ T cells, CD4^+^ T cells and CD8^+^ T cells at different time points pre and post HTx were assessed by flow cytometry in PBMC from n=17 HTx recipients; gating as shown in **Supplementary Figure 2A**. Absolute cell numbers of CD4^+^ and CD8^+^ T cells from **(D)** at time points *pre*, *T0* and *T24* were used to calculate the CD4^+^/CD8^+^ ratio from n=13 HTx recipients. **(F)** Whole blood samples from n=13 patients were quantified for absolute cell numbers of granulocytes at different time points pre and post HTx using TruCount^®^ analyses. Statistical analysis: A, D, F: 2-way-Anova was calculated for normally distributed data for time points *pre*, *T0* and *T24* and separately for time point *3wk* due to missing data. B, C, E: Repeated measure one-way ANOVA was calculated Data are shown as mean ± SEM. Significances are shown with gradations: p ≤ 0.05 (*), p ≤ 0.005 (**), p ≤ 0.0005 (***), p ≤ 0.0001 (****).

Immune phenotyping of recipient blood following HTx may provide insights in immune subset composition and therefore could be an important indicator for graft survival after surgery. Moreover, changes in the cytokine microenvironment depend on the composition of leukocyte subsets and *vice versa*. While previous, own work showed changing lymphocyte proportions after HTx (10), the interdependence of leukocyte subset composition and cytokine microenvironment has not been previously investigated in the context of HTx. To address the cellular changes induced by HTx, we analyzed the leukocyte dynamics before and after heart surgery and determined the relative frequencies and absolute cell numbers of the major immune cell subsets. We detected an absolute and relative increase in the monocyte count directly after HTx (*T0*, **Figures 2A, B**). Defining monocyte subsets (classical CD14^high^CD16^-^, intermediate CD14^high^CD16^+^, non-classical CD14^dim^CD16^+^, **Supplementary Figure 2B**), a relative increase of classical CD14^high^CD16^-^ monocytes as well as intermediate CD14^high^CD16^+^ monocytes was observed when comparing frequencies within all monocytes at *T0*, *T24* and *3wk* to *pre* HTx (**Figure 2B**). Simultaneously, a significant decrease in the proportion of non-classical CD14^dim^CD16^+^ monocytes from monocytes was detected after transplantation (**Figure 2B**). While the CD19^+^ B cell absolute count remained unchanged (**Figure 2A**), the absolute cell count of CD56^+^ NK cells was not changed directly after HTx, but decreased at *T24* (**Figure 2A**). In contrast, the relative proportion of total CD56^+^ NK cells strongly increased at *T0* compared to *pre*, and returned to base line level at *T24* (**Figure 2C**), probably as a result of a relative decrease in the frequency of CD3^+^ T cells within the CD45^+^ lymphocyte compartment (**Figure 2E**). Consistently, the absolute cell numbers of total CD3^+^ T cells and therefore CD4^+^ and CD8^+^ subsets decreased following transplantation (**Figure 2D**). While the frequencies of CD4^+^ T cells decreased at *T0*, a simultaneous increase in the CD8^+^ T cell proportion was noticed, resulting in an altered CD4^+^/CD8^+^ ratio at *T0* (**Figure 2E**; **Supplementary Figure 2A**), suggesting an effect of HTx primarily on the CD4^+^ T cell subset. The absolute count of granulocytes was also affected post HTx with higher cell counts at *T0* and *T24* (**Figure 2F**). Taken together, our results provide insights into the dynamics of the leukocyte composition after HTx, which may influence the cytokine/chemokine levels in HTx recipient blood.

### 3.3 CD14^high^CD16^+^ monocytes contribute to IL-10 production in HTx recipient blood

**Figure 3:**
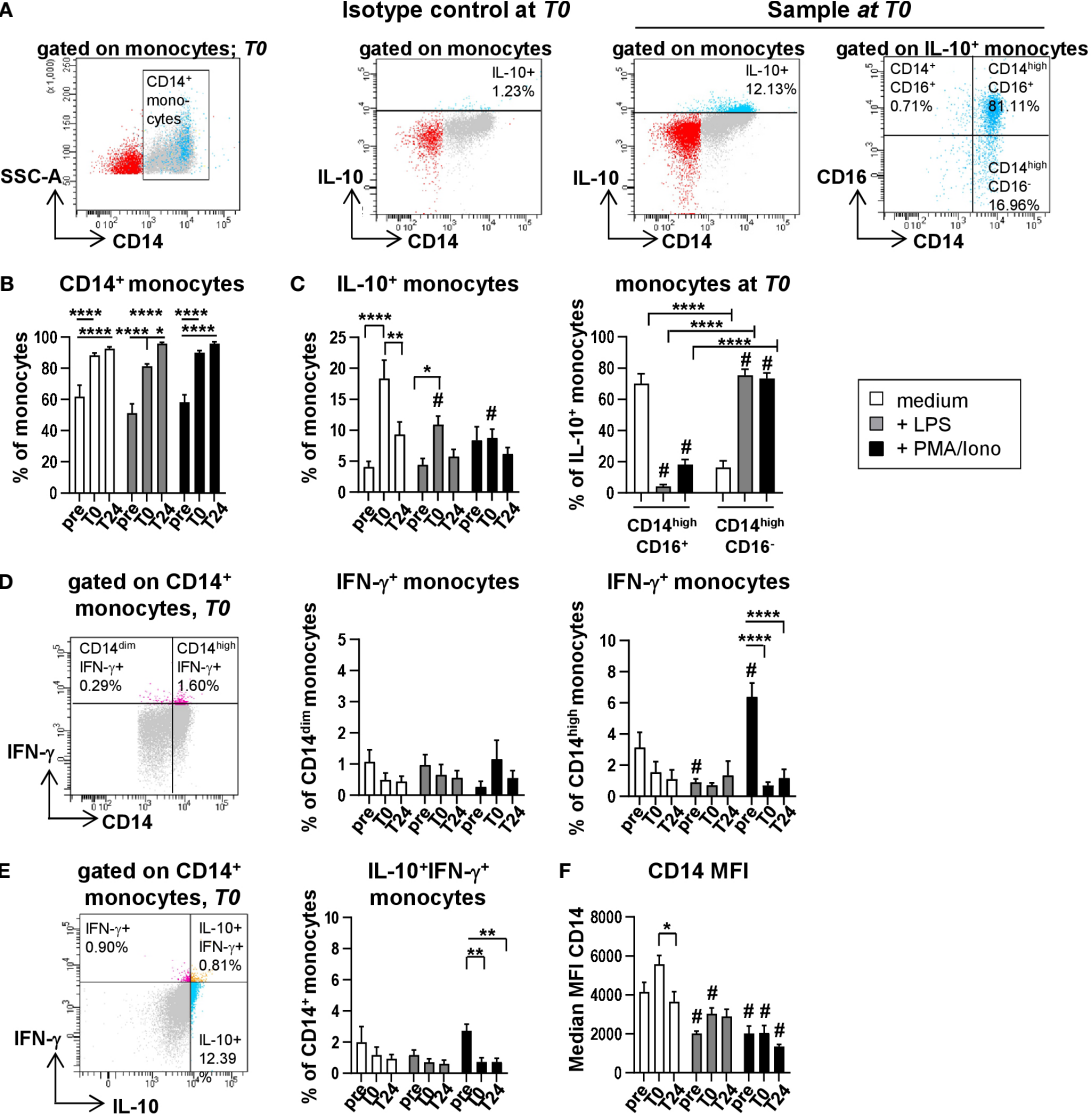
Monocytes spontaneously produce IL-10 without stimulation. Intracellular staining (ICS) for IL-10 and IFN-γ of unstimulated or LPS or PMA/Ionomycin stimulated PBMC from n=11 HTx patients at time points *pre*, *T0* and *T24*. *In vitro* stimulation was performed for 15h. Frequencies of IL-10^+^, IFN-γ^+^ as well as IL-10^+^IFN-γ^+^ double positive CD14^+^ monocytes were determined. Complete gating strategy is shown in **Supplementary Figures 3, 4**. **(A)** Representative FACS plots for gating of CD14^+^ monocytes, isotype control and exemplary intracellular IL-10 staining for gating of IL-10^+^ CD14^+^ monocytes and IL-10 producing CD14/CD16 monocyte subsets. Gating examples show unstimulated PBMC at *T0.* **(B)** Frequencies of CD14^+^ monocytes from time points *pre* to *T24* for unstimulated, LPS and PMA/Ionomycin stimulated PBMC (displayed as % from all monocytes). **(C)** Proportions of IL-10^+^ monocytes from gated CD14^+^ monocytes (left) and CD14^high^CD16^+^ or CD14^high^CD16^-^ monocytes at *T0* under unstimulated and stimulated conditions (right). **(D)** Exemplary gating shown at time point *T0*. Gating and frequencies of IFN-γ^+^ gated CD14^+^ monocytes at time points *pre* to *T24* for unstimulated, LPS and PMA/Ionomycin stimulated PBMC. Discrimination between CD14^dim^IFN-γ^+^ and CD14^high^IFN-γ^+^ monocytes. **(E)** Exemplary gating shown at time point *T0*. Representative gating and frequencies of IL-10^+^IFN-γ^+^ double positive CD14^+^ monocytes at time points *pre* to *T24* for unstimulated, LPS and PMA/Ionomycin stimulated PBMC. **(F)** Median fluorescence intensity (MFI) for CD14 from monocytes at different time points and stimulations is shown. Data were tested for normality; ICS data were analyzed with 2-way-Anova (n=11); data are shown as mean ± SEM; significances are shown with gradations: p ≤ 0.05 (*), p ≤ 0.005 (**), p ≤ 0.0001 (****); # is shown if stimulation differs from unstimulated at corresponding time point.

IL-10 exerts a cardio-protective function by inhibiting endothelial cell dependent T cell stimulation and human vascular smooth muscle cell proliferation and mediates immune-suppressive regulations in health and disease (11, 12). Even though we described the production of IL-10 protein after HTx previously (7), its cellular source has not been defined to date. In order to identify the cells responsible for the IL-10 peak in patient plasma directly after transplantation (*T0*), PBMC were stimulated with PMA/Ionomycin or LPS followed by intracellular staining (ICS) for IL-10 (**Figure 3A**). As the cross-talk between various immune cells subsets e.g. monocytes and T cells is important for their proper cytokine secretion, we triggered various immune cells via differential stimulation within the PBMC. For instance, PMA induces activation of protein kinase C and Ionomycin stimulates calcium release from the endoplasmatic reticulum they synergize in stimulating T cells (13, 14). LPS was used to address activation of myeloid cells via its ability to bind to Toll-Like receptor (TLR) 4 on monocytes and neutrophil granulocytes with subsequent intracellular signal transduction (14, 15).

Unstimulated PBMC from corresponding blood samples served as negative control. IL-10 production by major immune cell populations was determined by hierarchical gating and was found to be predominantly secreted by monocytes, but not by T, NK or B cells. While the frequencies of CD14^+^ monocytes increased over time (**Figures 3A, B** and **Supplementary Figure 3**) to comparable proportions shown in **Figures 2A, B**, highest intracellular frequencies of IL-10 were detected at *T0* in unstimulated monocytes (mean 18,38% of monocytes) and could not be further enhanced through LPS or PMA/Ionomycin stimulation (10,93% and 8,75% of monocytes, respectively) (**Figure 3C**). Moreover, unstimulated intermediate CD14^high^CD16^+^ monocytes were the main producers of IL-10 (**Figure 3C**), whereas upon stimulation with LPS or PMA/Ionomycin, IL-10 was predominantly produced by classical CD14^high^CD16^-^ monocytes (**Figure 3C**; **Supplementary Figure 4**). Interestingly, ICS analysis for IL-10 production by CD14^+^ monocytes reflected our previous findings at the protein and transcriptional level (**Figure 1**) with a peak at *T0* followed by a decrease at *T24* (**Figure 3C**). Both a raised count of absolute and relative monocytes post HTx and their high intracellular IL-10 signals indicate that monocytes are contributing to the high plasma level of IL-10 at *T0*. Of note, we observed elevated proportions of IL-10-producing monocytes at *T0* predominantly within unstimulated samples (**Figure 3C**), supporting our notion that an increase in intracellular IL-10 is most likely not the result of the stimulation with either LPS or PMA/Ionomycin but rather induced by the surgical intervention and linked to IRI along with the activation of various immunological relevant pathways leading to IL-10 production.

Furthermore, even though CD14^high^ monocytes produced IFN-γ following PMA/Ionomycin stimulation (**Figure 3D**), they did not simultaneously produce IL-10 (**Figure 3E**), suggesting the presence of monocytes with pro- and anti-inflammatory properties. Interestingly, the proportion of IFN-γ-producing monocytes within the CD14^high^ monocytes is decreased at *T0* and *T24* as compared to *pre* (**Figure 3D**), indicating that the IS affects the functional capacity of these cells to an anti-inflammatory state. Lastly, we detected elevated median fluorescence intensities (MFI) of CD14 within the unstimulated samples at *T0* (**Figure 3F**). However, this change in MFI was only transient as it significantly decreased at *T24*, further supporting our observation of an increase in both frequencies and absolute numbers of CD14^high^ monocytes at *T0*.

In conclusion, we identified intermediate CD14^high^CD16^+^ monocytes as major producers of IL-10 in peripheral blood of HTx recipients.

### 3.4 Following stimulation, T cells do not produce IL-10 but IFN-γ irrespective of immunosuppression

**Figure 4:**
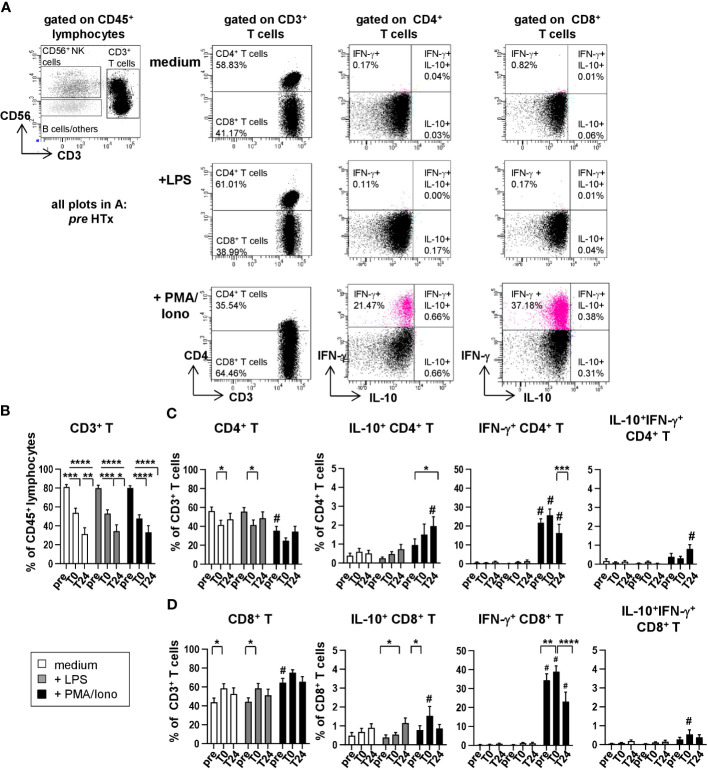
Upon stimulation with PMA/Ionomycin, T cells produce IFN-γ but no IL-10. Intracellular staining (ICS) for IL-10 and IFN-γ of unstimulated or LPS or PMA/Ionomycin stimulated PBMC from n=11 HTx patients at time points *pre*, *T0* and *T24*. *In vitro* stimulation was performed for 15h. Frequencies of IL-10^+^, IFN-γ^+^ as well as IL-10^+^IFN-γ^+^ double positive CD4^+^ and CD8^+^ T cells were determined. Complete gating strategy is shown in **Supplementary Figure 3**. **(A)** Representative FACS plots at time point *pre* for unstimulated, LPS and PMA/Ionomycin stimulated PBMC. CD3^+^ T cells were further discriminated into CD4^+^ and CD8^+^ T cells and frequencies of IL-10^+^ or IFN-γ^+^ single and IL-10^+^IFN-γ^+^ double positive CD4^+^ and CD8^+^ T cells were determined. **(B–D)** Frequencies of CD3^+^ T cells, CD4^+^ T cells and CD8^+^ T cells as well as IL-10^+^, IFN-γ^+^ as well as IL-10^+^IFN-γ^+^ double positive CD4^+^ and CD8^+^ T cells, respectively, in either unstimulated or stimulated conditions from time points *pre* to *T24*. Data were tested for normality and 2-way-Anova was calculated for n=11; data are shown as mean ± SEM. Significances are shown with gradations: p ≤ 0.05 (*), p ≤ 0.005 (**), p ≤ 0.0005 (***), p ≤ 0.0001 (****); # is shown if stimulation differs from unstimulated at corresponding time point.

Since we observed HTx related dynamics in the absolute and relative cell counts of CD3^+^ total T cells as well as CD4^+^ and CD8^+^ T cells (**Figure 2**), we analyzed if those dynamics translated into distinct cytokine production capabilities before and after HTx for *in vitro* stimulated T cells (**Figure 4A** and **Supplementary Figure 3**). Coherent with the absolute cell counts, percentages from CD3^+^ T cells within CD45^+^ lymphocytes decreased continuously after HTx (**Figure 4B**). Within CD3^+^ T cells, CD4^+^ T cells decreased and CD8^+^ T cells increased (**Figures 4C, D**), contributing to the altered CD4^+^/CD8^+^ T cell ratio described above (**Figure 2E**). As expected, we observed a small increase of IL-10-producing CD4^+^ and CD8^+^ T cells at very low frequencies after stimulating the PMBC with PMA/Ionomycin. However, because the proportion of IL-10-producing CD4^+^ and CD8^+^ T cells were minimal and their proportions were almost not detectable in unstimulated PBMC, both CD4^+^ and CD8^+^ T cells are most likely not responsible for the plasma IL-10 peak at *T0 in vivo.* In contrast, PMA/Ionomycin stimulation led to high percentages of IFN-γ-producing T cells, for both CD4^+^ and CD8^+^ subsets, gradual decrease between time points *T0* and *T24* (**Figures 4C, D**).

### 3.5 NK cells contribute to the pro-inflammatory IFN-γ cytokine milieu

**Figure 5:**
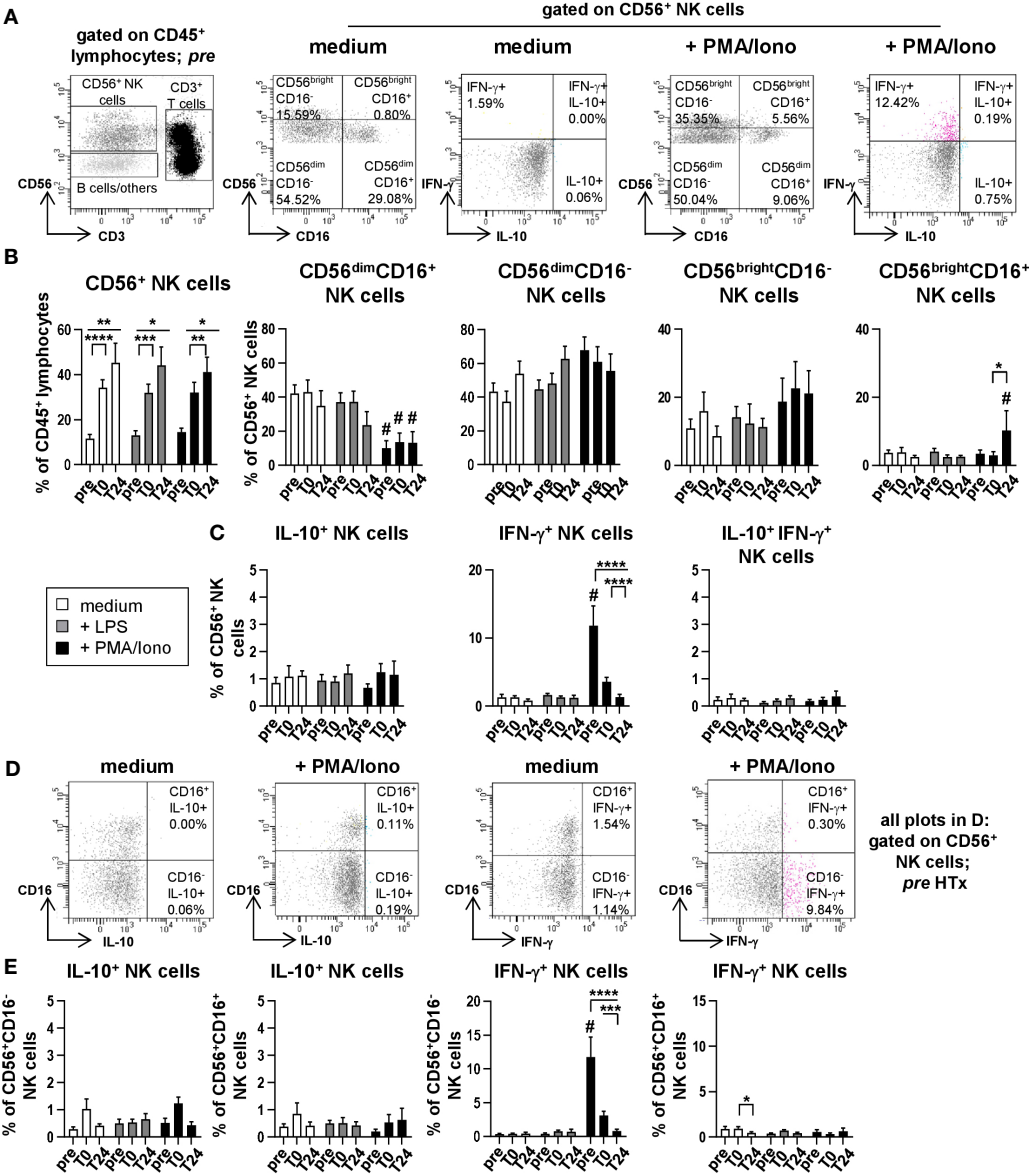
NK cells produce IFN-γ but no IL-10 after stimulation with PMA/Ionomycin. Intracellular staining (ICS) for IL-10 and IFN-γ of unstimulated or LPS or PMA/Ionomycin stimulated PBMC from n=11 HTx patients at time points *pre*, *T0* and *T24*. *In vitro* stimulation was performed for 15h. Frequencies of IL-10^+^, IFN-γ^+^ as well as IL-10^+^IFN-γ^+^ double positive CD56^+^ NK cells were determined. Complete gating strategy is shown in **Supplementary Figures 3, 5**. **(A)** Representative FACS plots at time point *pre* for unstimulated, and PMA/Ionomycin stimulated PBMC. NK cells were further characterized for expression of CD56^dim/bright^ and CD16^+/-^ and intracellular frequencies of IL-10^+^ or IFN-γ^+^ single and IL-10^+^IFN-γ^+^ double positive CD56^+^ NK cells were determined. **(B)** Frequencies of CD56^+^ NK and CD56^dim/bri^CD16^+/-^ NK subpopulations at time points *pre*, *T0* and *T24* for unstimulated, LPS and PMA/Ionomycin stimulated PBMC. **(C)** Frequencies of IL-10^+^, IFN-γ^+^ single and IL-10^+^IFN-γ^+^ double producing cells (as % of CD56^+^ NK cells). **(D)** Representative FACS plots of IL-10 and IFN-γ producing CD16^+/-^CD56^+^ NK cells at time point *pre* for unstimulated and PMA/Ionomycin stimulated PBMC. **(E)** Frequencies of IL-10 and IFN-γ producing CD16^+/-^CD56^+^ NK cells at time points *pre*, *T0* and *T24* for unstimulated, LPS and PMA/Ionomycin stimulated PBMC. Data were tested for normality and 2-way-Anova was calculated (n=11); data are shown as mean ± SEM. Significances are shown with gradations: p ≤ 0.05 (*), p ≤ 0.005 (**), p ≤ 0.0005 (***), p ≤ 0.0001 (****); # is shown if stimulation differs from unstimulated at corresponding time point.

To determine cytokine production by NK cell subsets, CD56^+^ NK cells (**Supplementary Figure 3**) were further discriminated into CD56^dim^CD16^+^, CD56^dim^CD16^-^, CD56^bright^CD16^-^ and CD56^bright^CD16^+^ NK cells (**Figures 5A, B**) and analyzed for intracellular IL-10 and IFN-γ production (**Figure 5C**). In line with previous own findings (9), we observed a strong increase in the proportion of CD56^+^ NK cells from gated CD45^+^ lymphocytes following HTx (unstimulated: *pre*: 11.55%; *T0*: 34.24%; *T24*: 45.30%; **Figure 5B**). Upon stimulation with PMA/Ionomycin, CD56^dim^ NK cells expressed lower levels of CD16 as compared to either unstimulated or LPS stimulated samples (**Figure 5B**; **Supplementary Figure 5**). This effect was not observed for CD56^bri^ NK cells (**Figure 5B**). We also detected high percentages of IFN-γ-producing CD56^+^ NK cells at *pre* after PMA/Ionomycin stimulation that were decreasing at later time points (**Figure 5C**) as previously described for T cells. Moreover, CD56^+^CD16^-^ NK cells were identified as the major IFN-γ-producing NK cell subset (**Figures 5D, E**). Thus, both T and NK cells are capable of producing IFN-γ before HTx but seems to be affected by the intraoperatively applied IS. We furthermore did not find compelling percentages of IL-10-producing CD20^+^ B cells (**Supplementary Figure 6**). Lastly, the preservation technique of the donor hearts did not influence cytokine production by monocytes, T and NK cells (**Supplementary Figure 7**).

Taken together the increased IL-10 concentrations measured in patient plasma directly after HTx are probably due to increasing frequencies and absolute cell counts of monocytes in peripheral blood of heart transplanted patients and a higher intrinsic capacity of these cells to produce IL-10.

## 4 Discussion

Despite the many improvements of surgical techniques in HTx as well as advances in drug monitored IS leading to prolonged overall survival, we are still lacking a precise understanding of the immune dynamics, i.e. the role of different leukocyte subsets in the clinical context of HTx and finally their contribution to the secretory immunomodulatory microenvironment. In human blood, different subsets of monocytes can be discriminated based on the expression of CD14 and CD16. According to the latest approved nomenclature, monocytes are classified into a major subset of CD14^high^CD16^-^ classical monocytes and two minor subpopulations: CD14^high^CD16^+^ intermediate and CD14^dim^CD16^+^ non-classical monocytes (16). Under physiological conditions, classical monocytes represent around 90% of blood-circulating monocytes, whereas intermediate and non-classical monocytes make up the remaining ~10% (17). While classical monocytes circulate in the blood for approximately one day, intermediate and non-classical monocytes have longer lifespans of around four and seven days, respectively, before differentiating into macrophages or dendritic cells (18). Thus, these two subsets could be more potent in eliciting systemic reactions following activation, as compared to classical monocytes. In consideration with those numbers, we found an astonishing overall increase of monocytes in peripheral blood of patients after HTx, and identified a distinct composition of monocytes, where intermediate monocytes increased up to 25% of all monocytes at 24 hours after transplantation *(T24)*. Concurrently, frequencies of non-classical CD14^low^CD16^+^, which are known to expand in the context of infections, especially in sepsis (19, 20), showed a persistent and significant decrease. Here, we identified intermediate monocytes as important cellular source of IL-10 in peripheral blood thereby confirming previous findings in healthy donors (21). Thus, not only in healthy donors but also in heart recipients, CD14^high^CD16^+^ intermediate monocytes are major producers of anti-inflammatory IL-10. The fact that we could not detect an additional increase in IL-10 production upon LPS stimulation by PBMCs isolated at *T0* as compared to unstimulated PBMC at this time point could be due to the Brefeldin A induced impaired activation of certain cytokines through LPS (22). Our data furthermore suggests that the *in vitro* stimulation shifts the IL-10 producing monocytes towards a CD14^high^CD16^-^ phenotype whereas under *in vivo* conditions CD14^high^CD16^+^ monocytes are the main producers of IL-10 by PBMC of HTx recipients.

Previously, monocytes have been described in connection to atherosclerosis and remodeling of the heart (23). More specifically, CD14^high^CD16^-^ classical monocytes served as a marker for poor prognosis in the context of organ failure (23–25), whereas in light of our study, CD14^high^CD16^+^ intermediate monocytes might play a beneficial role by producing anti-inflammatory IL-10, but not IFN-γ. Therefore, these cells, in addition to the IS given to transplant recipients, may contribute to shifting the balance to an anti-inflammatory state after HTx, as reflected by the decreased IFN-γ protein and mRNA in T cells at *T0* and *T24*. Adding to our hypothesis, we found that within our cohort, patients with severe primary graft dysfunction (PGD) showed significantly higher IL-10 concentrations directly post-operatively. Therefore, we suggest that within patients who later develop PGD, the early-onset of severe immune dynamics might lead to intensified activation of CD14^high^CD16^+^ monocytes with subsequent elevated IL-10 levels. Interestingly, though not significant (p = 0.09), females might show a stronger secretion of IL-10 compared to males. Nevertheless, we cannot exclude an additional contribution of blood granulocytes to the production of IL-10 after HTx that we could not assess in this study due to experimental settings (26). Granulocytes, in particular neutrophils, were shown to produce IL-10 during sepsis in mice (27). Thus the contribution of these cells to higher plasma IL-10 levels after HTx needs to be addressed in the future.

Importantly, immunosuppressive drugs did not affect the production of IL-10, but of IFN-γ, which was seen by comparison of basal levels of these cytokines at time point pre with time point T0 and T24 for the same patients. Consistently with our findings of increased absolute and relative monocyte counts in post HTx patient blood compared to *pre*, we previously measured higher concentrations of Monocyte Chemoattractant Protein-1 (MCP-1/CCL2) a key chemokine for migration and infiltration of monocytes/macrophages into the bloodstream and tissues (28) within HTx patients’ plasma (7). This observation suggests that the pro-inflammatory microenvironment in the graft following IRI may stimulate MCP-1 secretion thereby attracting monocyte migration from the bone marrow through the blood stream into the allograft. In line with our findings, upregulation of MCP-1 in the heart was found in models of myocardial infarction and reperfusion injury (29, 30) where human adult cardiac myocytes and human adult cardiac fibroblasts upregulated their MCP-1 expression following IRI (31).

The limitations of our study are a single center cohort of heart-transplanted patients with small patient number. In addition, the detection of IL-10 and/or the respective immune cell populations in endomyocardial biopsies would be desirable and could provide further insights especially when correlated to clinical outcomes.

Overall, our findings provide detailed insights into changes within the leukocyte compartment following HTx. Moreover, we provide evidence for an important role of CD14^high^CD16^+^ monocytes in the context of rejection *vs*. no rejection after solid organ transplantation of the human heart. Considering the limited long-term survival of patients after HTx (32), it is of vital importance to unravel promising strategies to overcome this obstacle. Analogues to findings in IL-10 *ex-vivo* gene transfer in the context of lung transplantation (33), induction of IL-10-producing monocytes intraoperatively could represent the beginning of a novel therapeutic approach in order to prevent early establishment of a pro-inflammatory milieu, which leads to long-term rejection potential or the induction of chronic allograft vasculopathy. Monocytes could, therefore, be a key player in the future of immune transplant management and clinical outcome of transplanted patients.

## Data availability statement

The original contributions presented in the study are included in the article/**Supplementary Material**. Further inquiries can be directed to the corresponding author.

## Ethics statement

The studies involving humans were approved by Ethikkommission der Medizinischen Hochschule Hannover. The studies were conducted in accordance with the local legislation and institutional requirements. The participants provided their written informed consent to participate in this study.

## Author contributions

KL: Investigation, Methodology, Data curation, Formal Analysis, Visualization, Writing – original draft. EC: Investigation, Data curation, Formal Analysis, Supervision, Visualization, Writing – original draft. JFK: Investigation, Data curation, Formal Analysis, Validation, Visualization, Writing – original draft. BW: Methodology, Resources. JI: Investigation. NL: Investigation. FI: Data curation, Methodology, Resources. KB: Investigation, Methodology, Validation. JK: Investigation, Methodology, Validation. SI: Investigation, Methodology. SVR: Methodology. JS: Methodology. AKK: Investigation. AH: Methodology. GW: Methodology, Writing – original draft. CF: Conceptualization, Data curation, Formal Analysis, Funding acquisition, Project administration, Supervision, Validation, Visualization, Writing – original draft.

## References

[1] Chichelnitskiy E Himmelseher B Bachmann M Pfeilschifter J Mühl H. Hypothermia promotes interleukin-22 expression and fine-tunes its biological activity. Front Immunol (2017) 8:742. doi: 10.3389/fimmu.2017.00742 28706520 PMC5489602

[2] El-Sawy T Fahmy NM Fairchild RL. Chemokines: Directing leukocyte infiltration into allografts. Curr Opin Immunol (2002) 14(5):562–8. doi: 10.1016/S0952-7915(02)00382-5 12183154

[3] Nakamura Y Saito S Miyagawa S Yoshikawa Y Hata H Yoshioka D. Perioperative ischaemic reperfusion injury and allograft function in the early post-transplantation period. Interact Cardiovasc Thorac Surg (2019) 29(2):230–6. doi: 10.1093/icvts/ivz086 30919896

[4] Spartalis M Spartalis E Tzatzaki E Tsilimigras DI Moris D Kontogiannis C. Cardiac allograft vasculopathy after heart transplantation: Current prevention and treatment strategies. Eur Rev Med Pharmacol Sci (2019) 23(1):303–11. doi: 10.26355/eurrev_201901_16777 30657571

[5] Li W Fu F Lu L Narula SK Fung JJ Thomson AW. Differential effects of exogenous Interleukin-10 on cardiac allograft survival 1,2: Inhibition of rejection by recipient pretreatment reflects impaired host accessory cell function. Transplantation (1999) 68(9):1402. doi: 10.1097/00007890-199911150-00029 10573082

[6] Verma R Balakrishnan L Sharma K Khan AA Advani J Gowda H. A network map of interleukin-10 signaling pathway. J Cell Commun Signal (2016) 10(1):61–7. doi: 10.1007/s12079-015-0302-x PMC4850137 26253919

[7] Ledwoch N Wiegmann B Chichelnitskiy E Wandrer F Kühne JF Beushausen K. Identification of distinct secretory patterns and their regulatory networks of ischemia versus reperfusion phases in clinical heart transplantation. Cytokine (2022) 149:155744. doi: 10.1016/j.cyto.2021.155744 34649160

[8] Kobashigawa J Zuckermann A Macdonald P Leprince P Esmailian F Luu M. Consensus Conference participants. Report from a consensus conference on primary graft dysfunction after cardiac transplantation. J Heart Lung Transplant (2014) 33(4):327–40. doi: 10.1016/j.healun.2014.02.027 24661451

[9] Cossarizza A Chang H Radbruch A Abrignani S Addo R Akdis M. Guidelines for the use of flow cytometry and cell sorting in immunological studies (third edition). Eur J Immunol (2021) 51(12):2708–3145. doi: 10.1002/eji.202170126 34910301 PMC11115438

[10] Iske J Wiegmann B Ius F Chichelnitskiy E Ludwig K Kühne JF. Immediate major dynamic changes in the T- and NK-cell subset composition after cardiac transplantation. Eur J Immunol (2023) 53(7):e2250097. doi: 10.1002/eji.202250097 37119053

[11] Gleissner CA Zastrow A Klingenberg R Kluger MS Konstandin M Celik S. IL-10 inhibits endothelium-dependent T cell costimulation by up-regulation of ILT3/4 in human vascular endothelial cells. Eur J Immunol (2007) 37(1):177–92. doi: 10.1002/eji.200636498 17163451

[12] Selzman CH McIntyre RC Jr. Shames BD Whitehill TA Banerjee A Harken AH. Interleukin-10 inhibits human vascular smooth muscle proliferation. J Mol Cell Cardiol (1998) 30(4):889–96. doi: 10.1006/jmcc.1998.0642 9602438

[13] Ai W Li H Song N Li L Chen H. Optimal method to stimulate cytokine production and its use in immunotoxicity assessment. Int J Environ Res Public Health (2013) 10(9):3834–42. doi: 10.3390/ijerph10093834 PMC3799516 23985769

[14] Mandala W Harawa V Munyenyembe A Soko M Longwe H. Optimization of stimulation and staining conditions for intracellular cytokine staining (ICS) for determination of cytokine-producing T cells and monocytes. Curr Res Immunol (2021) 2:184–93. doi: 10.1016/j.crimmu.2021.10.002 PMC9040130 35492400

[15] Rossol M Heine H Meusch U Quandt D Klein C Sweet MJ. LPS-induced cytokine production in human monocytes and macrophages. Crit Rev Immunol (2011) 31(5):379–446. doi: 10.1615/CritRevImmunol.v31.i5.20 22142165

[16] Ziegler-Heitbrock L Ancuta P Crowe S Dalod M Grau V Hart DN. Nomenclature of monocytes and dendritic cells in blood. Blood (2010) 116(16):e74–80. doi: 10.1182/blood-2010-02-258558 20628149

[17] Wong KL Tai JJ Wong W Han H Sem X Yeap WH. Gene expression profiling reveals the defining features of the classical, intermediate, and nonclassical human monocyte subsets. Blood (2011) 118(5):16. doi: 10.1182/blood-2010-12-326355 21653326

[18] Patel AA Zhang Y Fullerton JN Boelen L Rongvaux A Maini AA. The fate and lifespan of human monocyte subsets in steady state and systemic inflammation. J Exp Med (2017) 214(7):1913–23. doi: 10.1084/jem.20170355 PMC5502436 28606987

[19] Nockher WA Scherberich JE. Expanded CD14^+^ CD16^+^ monocyte subpopulation in patients with acute and chronic infections undergoing hemodialysis. Infect Immun (1998) 66(6):2782–90. doi: 10.1128/IAI.66.6.2782-2790.1998 PMC108270 9596748

[20] Gainaru G Papadopoulos A Tsangaris I Lada M Giamarellos-Bourboulis EJ Pistiki A. Increases in inflammatory and CD14dim/CD16pos/CD45pos patrolling monocytes in sepsis: Correlation with final outcome. Crit Care (2018) 22(1):56. doi: 10.1186/s13054-018-1977-1 29499723 PMC5834896

[21] Skrzeczyńska-Moncznik J Bzowska M Loseke S Grage-Griebenow E Zembala M Pryjma J. Peripheral blood CD14high CD16+ monocytes are main producers of IL-10. Scandinavian J Immunol (2008) 67(2):152–9. doi: 10.1111/j.1365-3083.2007.02051.x 18201370

[22] Hong S Hwang I Gim E Yang J Sangjun Park S Yoon SH. Brefeldin A-sensitive ER-golgi vesicle trafficking contributes to NLRP3-dependent caspase-1 activation. FASEB J (2019) 33(3):4547–58. doi: 10.1096/fj.201801585R 30592629

[23] Wildgruber M Aschenbrenner T Wendorff H Czubba M Glinzer A Haller B. The “Intermediate” CD14^++^CD16^+^ monocyte subset increases in severe peripheral artery disease in humans. Sci Rep (2016) 6(1):39483. doi: 10.1038/srep39483 27991581 PMC5171878

[24] Abeles RD McPhail MJ Sowter D Antoniades CG Vergis N Vijay GKM. CD14, CD16 and HLA-DR reliably identifies human monocytes and their subsets in the context of pathologically reduced HLA-DR expression by CD14hi/CD16neg monocytes: Expansion of CD14hi/CD16pos and contraction of CD14lo/CD16pos monocytes in acute liver failure. Cytometry Part A. (2012) 81A(10):823–34. doi: 10.1002/cyto.a.22104 22837127

[25] Zhang R Shi J Zhang R Ni J Habtezion A Wang X. Expanded CD14hiCD16– immunosuppressive monocytes predict disease severity in patients with acute pancreatitis. J Immunol (2019) 202(9):2578–84. doi: 10.4049/jimmunol.1801194 30894427

[26] Kuhns DB Priel DAL Chu J Zarember KA. Isolation and functional analysis of human neutrophils. Curr Protoc Immunol (2015) 111(1):7.23.1–7.23.16. doi: 10.1002/0471142735.im0723s111 PMC4668800 26528633

[27] Kasten KR Muenzer JT Caldwell CC. Neutrophils are significant producers of IL-10 during sepsis. Biochem Biophys Res Commun (2010) 393(1):28–31. doi: 10.1016/j.bbrc.2010.01.066 20097159 PMC2830356

[28] Deshmane SL Kremlev S Amini S Sawaya BE. Monocyte chemoattractant protein-1 (MCP-1): An overview. J Interferon Cytokine Res (2009) 29(6):313–26. doi: 10.1089/jir.2008.0027 PMC2755091 19441883

[29] Kumar AG Ballantyne CM Michael LH Kukielka GL Youker KA Lindsey ML. Induction of monocyte chemoattractant protein-1 in the small veins of the ischemic and reperfused canine myocardium. Circulation (1997) 95(3):693–700. doi: 10.1161/01.CIR.95.3.693 9024159

[30] Dewald O Ren G Duerr GD Zoerlein M Klemm C Gersch C. Of mice and dogs: Species-specific differences in the inflammatory response following myocardial infarction. Am J Pathol (2004) 164(2):665–77. doi: 10.1016/S0002-9440(10)63154-9 PMC1602262 14742270

[31] Hohensinner PJ Kaun C Rychli K Ben-Tal Cohen E Kastl SP Demyanets S. Monocyte chemoattractant protein (MCP-1) is expressed in human cardiac cells and is differentially regulated by inflammatory mediators and hypoxia. FEBS Lett (2006) 580(14):3532–8. doi: 10.1016/j.febslet.2006.05.043 16730716

[32] Lund L Edwards L Kucheryavaya A Benden C Christie JD Dipchand AI. The registry of the international society for heart and lung transplantation: Thirty-first official adult heart transplant Report—2014; focus theme: Retransplantation. J Heart Lung Transplant (2014) 33(10):996–1008. doi: 10.1016/j.healun.2014.08.003 25242124

[33] Cypel M Liu M Rubacha M Yeung JC Hirayama S Anraku M. Functional repair of human donor lungs by IL-10 gene therapy. Sci Trans Med (2009) 1(4):4ra9. doi: 10.1126/scitranslmed.3000266 20368171

