# Supplementary material for: CD14highCD16+ monocytes are the main producers of Interleukin-10 following clinical heart transplantation
Source: Front Immunol. 2023 Oct 23;14:1257526. doi: 10.3389/fimmu.2023.1257526 (PMC10627027; doi:10.3389/fimmu.2023.1257526)
Supplement: Supplementary file 1 [file DataSheet_1.pdf]

Supplementary Material

$CD14^{high}CD16^{+}$ monocytes are the main producers of Interleukin-10 following clinical heart transplantation

Kristina Ludwig, Evgeny Chichelnitskiy, Jenny F. Kühne, Bettina Wiegmann, Jasper Iske, Nadine Ledwoch, Fabio Ius, Kerstin Beushausen, Jana Keil, Susanne Iordanidis, Sebastian V. Rojas, Jawad Salman, Ann-Kathrin Knoefel, Axel Haverich, Gregor Warnecke and Christine S. Falk*

*** Correspondence:** Christine S. Falk: falk.christine@mh-hannover.de

Supplemetary Figure 1: Experimental set up for PBMC from patients at represented time points.

Supplemetary Figure 2: Gating strategy for flow cytometric analysis of cell surface stained PBMC.

Supplemetary Figure 3: Gating strategy for intracellular stained PBMC.

Supplemetary Figure 4: Gating strategy for the identification of $IL-10^{+}$ monocyte subsets.

Supplemetary Figure 5: LPS stimulation does not induce cytokine production by NK cells.

Supplemetary Figure 6: B cells do not produce intracellular IL-10 in HTx patients.

Supplemetary Figure 7: Cytokine secretion by monocytes, T and NK cells does not differ in ESHP and SOC preserved donor hearts.

Supplementary Table 1: List of fluorescently labelled antibodies used for cell surface staining and intracellular staining for flow cytometric analyses.

Supplementary Figures

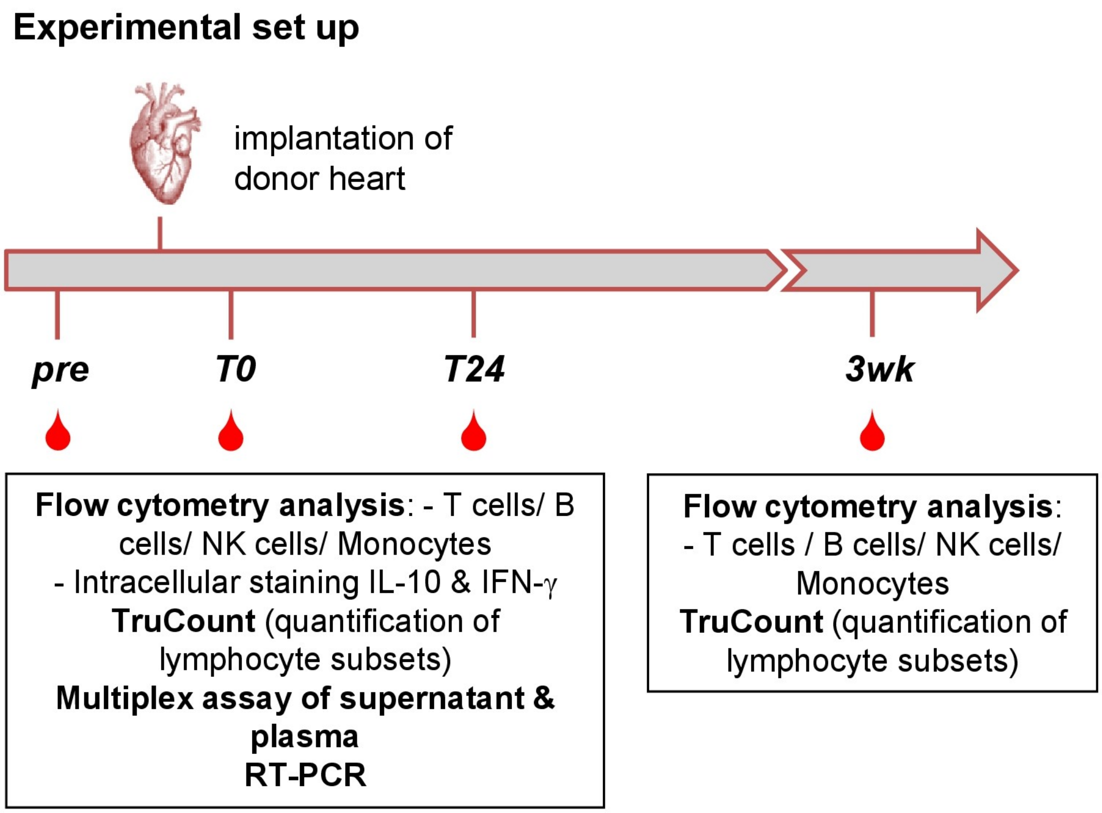

Supplementary Figure 1: Experimental set up for PBMC from patients at represented time points.

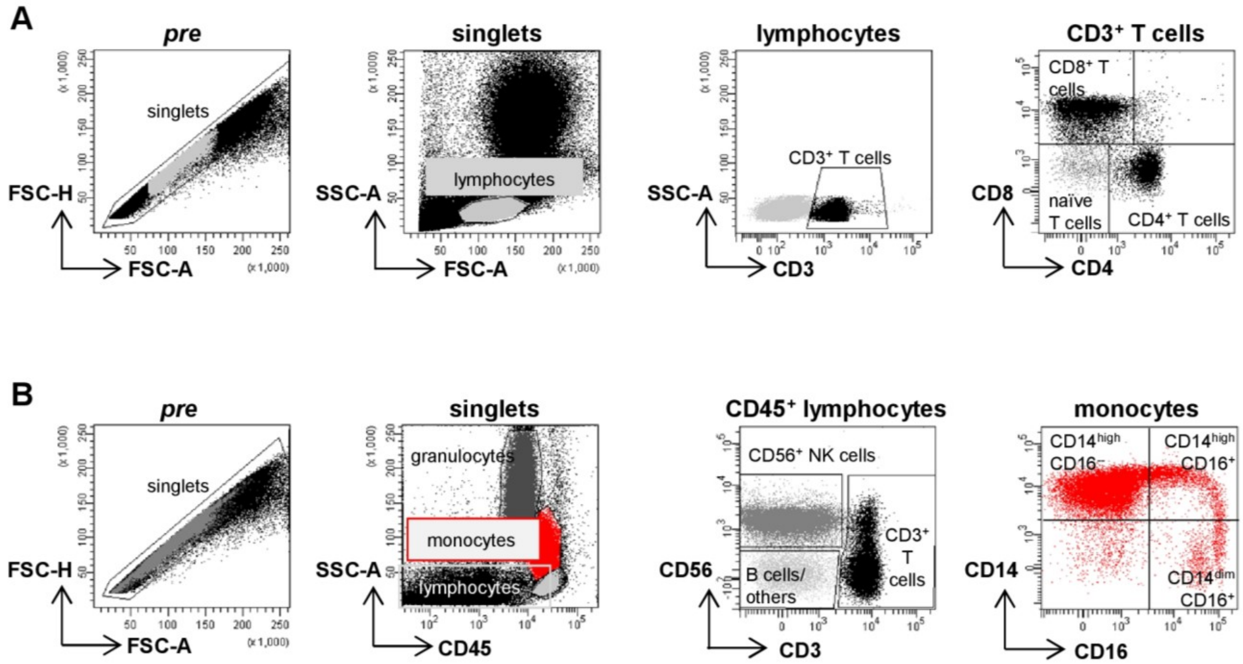

Supplementary Figure 2: Gating strategy for flow cytometric analysis of cell surface stained PBMC. Exemplary FACS plots for PBMC at time point *pre* after staining with fluorescently labelled antibodies as stated in Table S1. **(A)** Strategy for gating $CD3^+$ T cells. Cell singlets were further gated for lymphocytes based on FSC-A vs. SSC-A. In the next step, $CD3^+$ T cells were identified and these were further subdivided into $CD4^+$ and $CD8^+$ T cells, respectively. $CD4^-CD8^-$ were considered as naïve T cells. **(B)** Strategy for gating of monocytes and lymphocyte subsets. Single cells were further discriminated into $CD45^+$ lymphocytes, monocytes and granulocytes based on CD45 vs. SSC-A. From $CD45^+$ lymphocytes, $CD56^+$ NK cells, $CD3^+$ T cells and B cells/others were gated. Monocytes were further gated for $CD14^{high}CD16^-$, $CD14^{high}CD16^+$ and $CD14^{dim}CD16^+$ monocyte subsets.

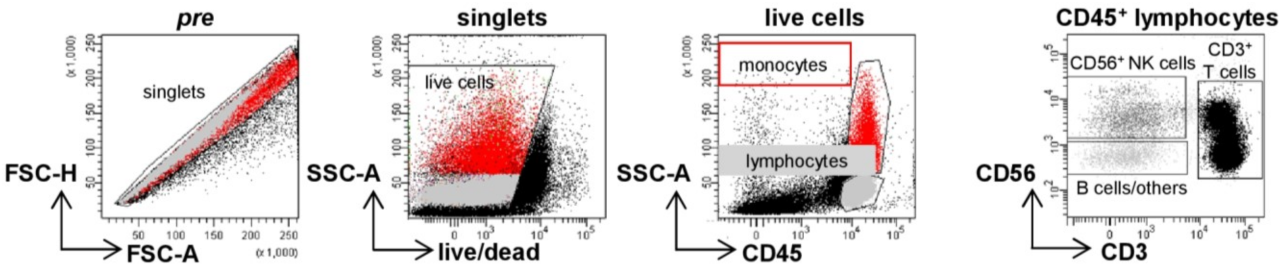

Supplementary Figure 3: **Gating strategy for intracellular stained PBMC.** Exemplary gating strategy for unstimulated PBMC at time point *pre* after staining with fluorescently labelled antibodies as stated in Table S1. Cell singlets were further gated for live cells with following gating for $CD45^+$ lymphocytes and monocytes. From $CD45^+$ lymphocytes, $CD56^+$ NK cells, $CD3^+$ T cells and B cells/others ($CD3^-CD56^-$) were gated.

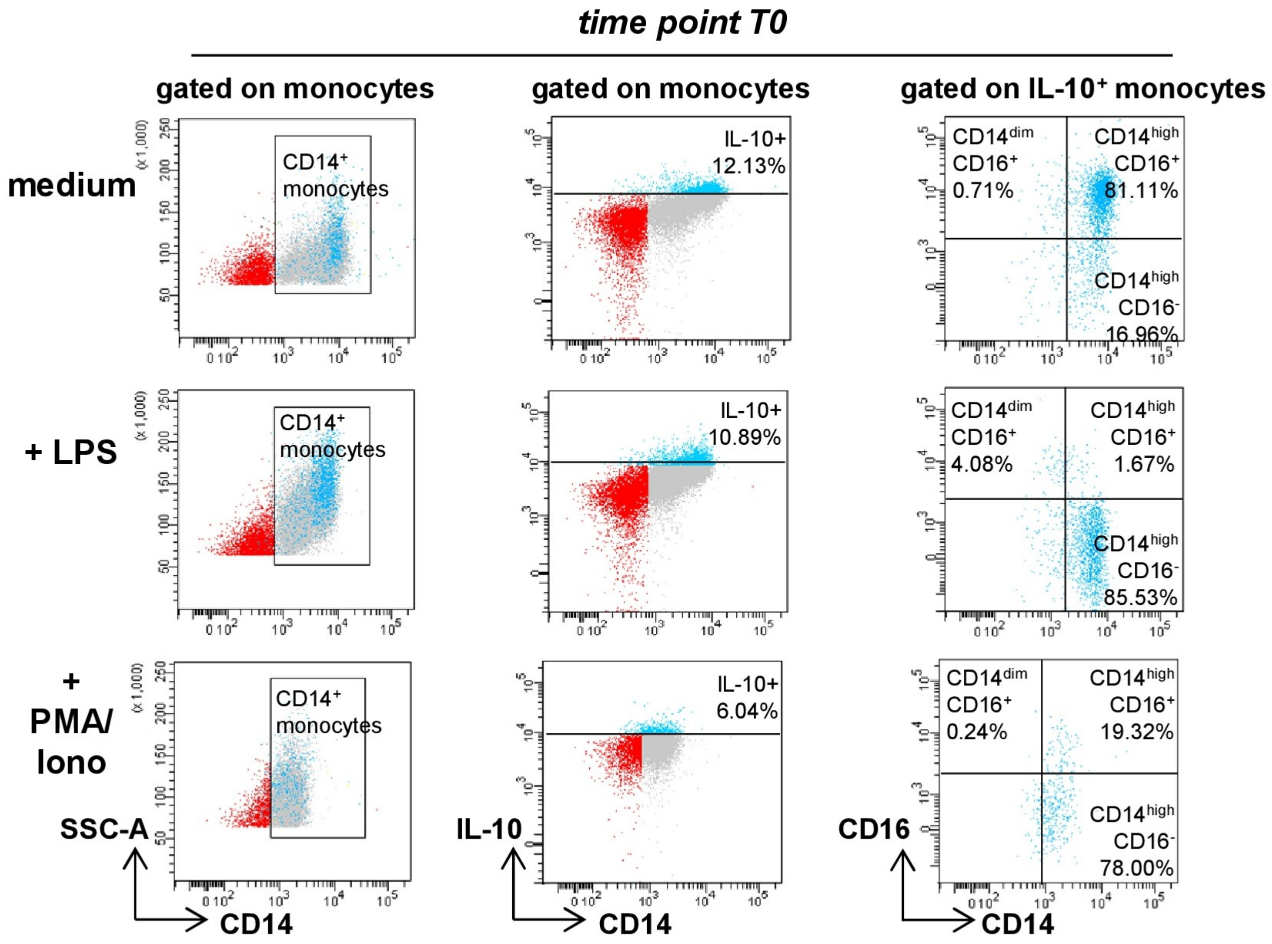

Supplementary Figure 4: **Gating strategy for the identification of IL-10$^+$ monocyte subsets.** Representative FACS plots for unstimulated, LPS and PMA/Ionomycin stimulated PBMC at time point *T0*. Following *in vitro* stimulation for 15h, PBMC were stained with a cell viability dye as well as fluorescently labelled antibodies (surface and intracellular) as described in Material and methods. Following the gating described in Supplementary Figure 3, CD14$^+$ monocytes were further analyzed for intracellular IL-10 production and finally the phenotype of IL-10$^+$CD14$^+$ monocytes was identified (CD14$^{high/dim}$CD16$^{+/-}$).

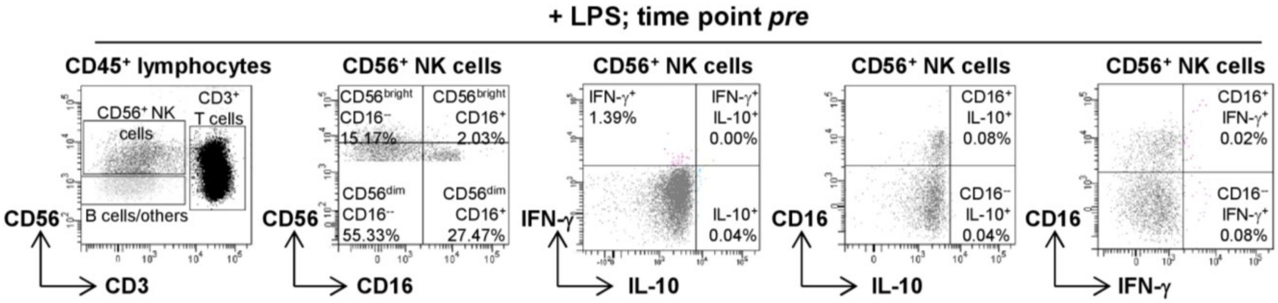

Supplementary Figure 5: **LPS stimulation does not induce cytokine production by NK cells.** Gating examples of LPS stimulated PBMC at *T0* following gating strategy as shown in Supplementary Figure 3 for the identification of IFN-γ and IL-10 producing $CD56^{+}CD16^{+/-}$ NK cell subsets.

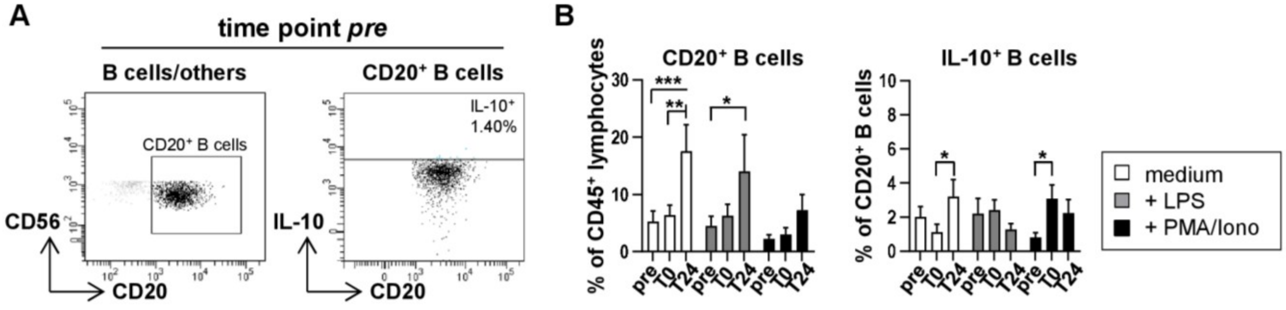

Supplementary Figure 6: B cells do not produce intracellular IL-10 in HTx patients. Following *in vitro* stimulation for 15h, PBMC were stained with a cell viability dye as well as fluorescently labelled antibodies (surface and intracellular) as described in Material and methods. B cells were identified as described in Supplementary Figure 3. **(A)** Representative FACS plots of unstimulated PBMC at *T0* for gating of IL-10 producing B cells. CD20$^+$ B cells were selected from total B cells (CD3$^-$CD56$^-$) and subsequently gated for IL-10$^{+/-}$CD20$^+$ B cells. **(B)** Frequencies of CD20$^+$ B cells (as % of CD45$^+$ lymphocytes) and IL-10$^+$CD20$^+$ B cells (% of CD20$^+$ B cells) at time points *pre, T0* and *T24* for unstimulated, LPS and PMA/Ionomycin stimulated PBMC of n=11 HTx patients. Data were tested for normality and 2-way-Anova was calculated (n=11); data are shown as mean ± SEM. Significances are shown with gradations: p≤0.05 (*), p≤0.005 (**), p≤0.0005 (***), p≤0.0001 (****); # is shown if stimulation differs from unstimulated at corresponding time point.

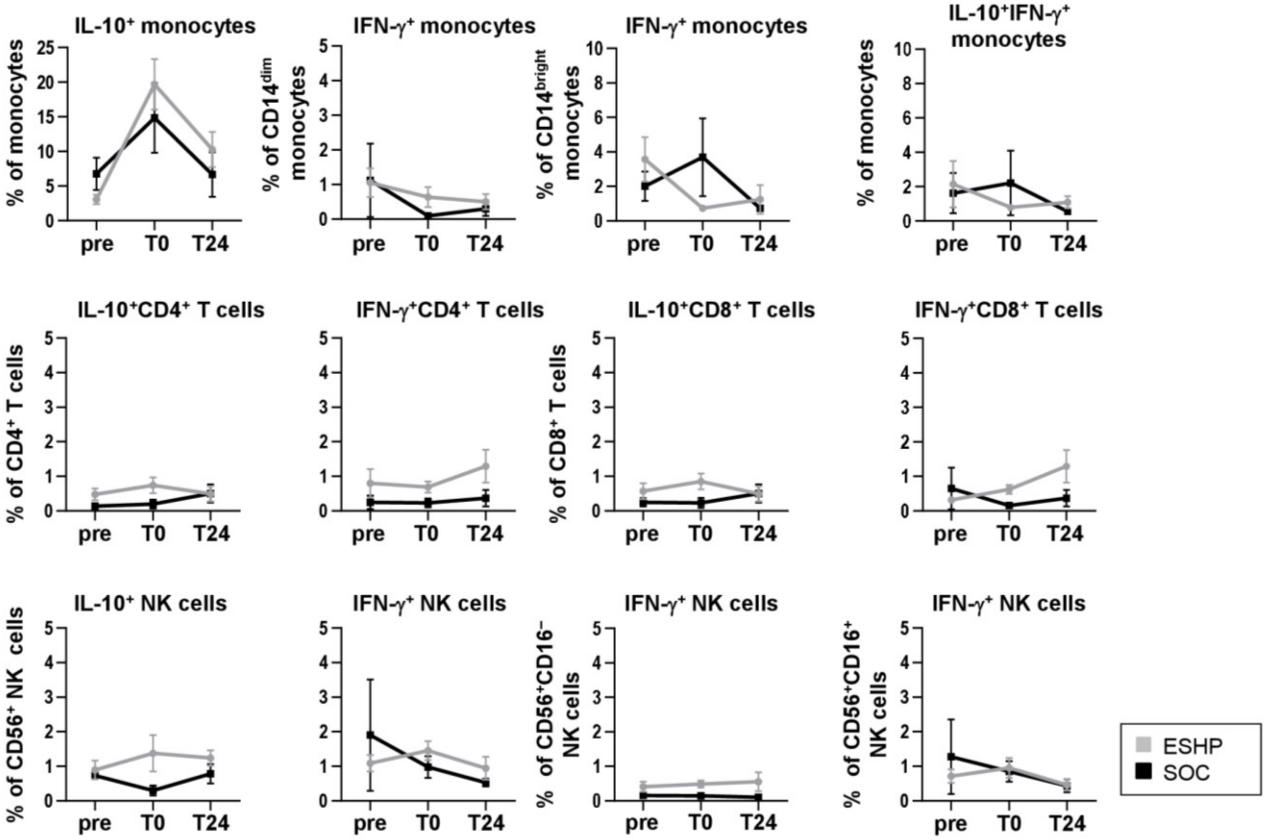

Supplementary Figure 7: Cytokine secretion by monocytes, T and NK cells does not differ in ESHP and SOC preserved donor hearts. Frequencies of IL-10$^+$ and IFN-γ$^+$ monocyte-, T- cell and NK cell subsets of unstimulated PBMC from n=8 ESHP patients and n=3 SOC patients at time points *pre*, *T0* and *T24.* For gating strategy, please see Figures 3-5 and Supplementary Figures 2 and 3. 2-way-ANOVA was calculated with following Sidak's test for multiple comparison. Data are shown as mean ± SEM. Significances are shown with gradations: p≤0.05 (*), p≤0.005 (**), p≤0.0005 (***), p≤0.0001 (****).

Supplementary Tables

Supplementary Table 1. List of fluorescently labelled antibodies used for cell surface staining and intracellular staining for flow cytometric analyses.

Antigen	Fluorochrome	Clone	Manufacturer
**CD3**	APC-H7	SK7	BD Biosciences
**CD3**	PerCP	SK7	BD Biosciences
**CD3**	Horizon-V500	UCHT1	BD Biosciences
**CD3**	PE-Cy7	UCHT1	BioLegend
**CD4**	PerCP	SK3	BD Biosciences
**CD4**	PerCP-Cy5.5	RPA-T4	BioLegend
**CD6**	FITC	M-T605	BD Biosciences
**CD8**	APC-H7	SK1	BD Biosciences
**CD14**	PE-Cy7	MφP9	BD Biosciences
**CD14**	FITC	M5E2	BD Biosciences
**CD16**	APC	B73.1	BD Biosciences
**CD16**	APC-Cy7	3G8	BD Biosciences
**CD19**	PerCP	SJ25C1	BD Biosciences
**CD20**	APC-H7	L27	BD Biosciences
**CD20**	Horizon-V450	L27	BD Biosciences
**CD45**	Alexa Fluor 700	HI30	BioLegend
**CD45**	APC-H7	2D1	BD Biosciences
**CD45**	Alexa Fluor 700	HI30	BioLegend
**CD56**	PE	B159	BD Biosciences
**CD56**	BV510	NCAM16.2	BD Biosciences
**Intracellular staining**			
**IL-10**	PE	JES3-9D7	BioLegend
**Isotype control**	PE	7T4-1F5	Beckman Coulter
**IFN-γ**	APC	4S.B3	BioLegend
**Isotype control**	Alexa Fluor 647	MOPC-21	BD Biosciences
